# Evaluating interventions for informed consent for surgery (ICONS): Protocol for the development of a core outcome set

**DOI:** 10.1186/s13063-018-2986-8

**Published:** 2018-11-06

**Authors:** Liam J. Convie, Scott McCain, Jeffrey Campbell, Stephen J. Kirk, Mike Clarke

**Affiliations:** 1 Department of General Surgery, Ulster Hospital, Dundonald, UK; 2 Centre for Public Health, Queen’s University Belfast, Belfast, UK

**Keywords:** Core outcome sets, Consensus methods, Informed consent, Surgery

## Abstract

**Background:**

The concept of informed consent is fundamental to medical practice. Shortcomings in the process can lead to patient complaints, litigation, unmet expectations and poor outcomes. Consent research has focused on developing tools to improve patient recall and understanding. However, the definitions, methods of measurement and timing of measurement vary widely across the studies that have been done. Although a Cochrane review has reported that many of these interventions appear to work, the high level of heterogeneity in outcome reporting prevents the identification of those interventions that work best and why they do so. It is also not clear which outcomes are most important to each party involved in the consent process and why.

**Methods/design:**

This project will develop a core outcome set for assessing the effects of interventions aimed at improving informed consent for surgery and other invasive procedures for adult patients with the capacity to consent for themselves. We will conduct a systematic review of the qualitative and quantitative literature to identify outcomes used to date in consent research and map these into domains. A series of semi-structured key stakeholder interviews will also be used to identify relevant outcomes. These processes will produce a list of potential outcomes for assessing the effects of interventions to improve consent, which will be refined through an international Delphi survey and consensus webinars involving key stakeholders to produce the core outcome set.

**Discussion:**

The ICONS study aims to develop a core outcome set for use in trials and reviews of interventions designed to improve the informed consent process for surgery and other invasive procedures. Our aim is that this core outcome set will reduce the level of selection and reporting bias in consent research and help clinicians to compare tools to improve consent.

**Electronic supplementary material:**

The online version of this article (10.1186/s13063-018-2986-8) contains supplementary material, which is available to authorized users.

## Background

Consent represents one of the cornerstones of good medical practice as outlined by the General Medical Council [1]. Consent is considered to be valid when a competent patient knows and understands the potential harms, benefits and alternatives of the intervention and uses that information to make their decision regarding treatment and communicates their decision free from coercion [2]. The consent process continues to be a significant challenge for medical practice. It has come to prominence again in the Montgomery case [3].

In 1999, Mrs Montgomery was an expectant mother from Lanarkshire in the United Kingdom. Mrs Montgomery also had diabetes mellitus. Women with diabetes are at higher risk of having larger than normal babies, which increases the risk of shoulder dystocia. Mrs Montgomery had not been informed of the risk of shoulder dystocia, as it had been deemed by her obstetrician that the risk of shoulder dystocia leading to a serious problem was very small. Mrs Montgomery claimed that if she had known about the risks of shoulder dystocia, she would have requested a caesarean section. The shoulder of Mrs Montgomery’s baby became stuck during childbirth, resulting in 12 min of cerebral hypoxia, cerebral palsy and quadriplegia.

In 2015, the Supreme Court ruling explained the materiality of risk. That is, if a reasonable patient in Mrs Montgomery’s position had been informed of the risk of obstructed labour, and the possibility of caesarean section as an alternative method of delivery to avoid that complication, she would have likely attached significance to that information.

This ruling represents a significant change in UK medical law. Previously, the level of disclosure expected from medical professionals had been determined by a reasonable body of contemporary medical opinion [4, 5]. However, following the Montgomery ruling, that standard of disclosure ought to be determined by what is expected of a ‘reasonable patient’. There may indeed be a third level of disclosure. Mazur proposes the ‘subjective patient standard’. That is to say, the clinician should supply the information that the particular patient in question would want to know [6]. This clearly would have significant implications for clinical practice.

Obtaining informed consent involves a clinician having a discussion with the patient. Through this conversation, the clinician will outline the nature of the proposed intervention, the potential harms and benefits, and the alternatives. They will also provide an opportunity for the patient to ask questions about the treatment. This process may take place in one session or across a number of meetings and is often augmented with the use of written, web-based or video information.

Attempts have been made to improve the consent process by various means, some of which have been tested in research studies. Stacey and colleagues’ Cochrane review of decision aids for patients facing health treatment and screening decisions found high quality evidence to suggest that decision aids improve patients’ knowledge regarding their options and reduce their level of decisional conflict [7]. However, the review was limited by the large amount of heterogeneity in the outcomes used to determine the effect of the decision aids in the consent process. Similarly, a Cochrane review of interventions to promote informed consent for invasive procedures concluded that interventions to improve consent consistently improved patient knowledge [8]. However, although knowledge is clearly an important step in achieving informed consent, the authors of the review found that the tremendous heterogeneity in outcomes measured and how they were recorded made comparisons between studies difficult. They suggested that future work ought to focus on developing a consensus on what outcomes should be measured to determine the quality of informed consent and to use or develop validated and standardised tools to measure those outcomes. For example, in addition to patient recall of consent-related information, outcomes such as understanding, decisional conflict, deliberation, decisional regret, anxiety and satisfaction, among others, might be considered.

Core outcome sets aim to define a set of outcomes that should be considered essential in the evaluation and reporting of a particular intervention or condition [9]. There are well-defined guidelines with a growing evidence base to support the use of core outcome sets and the methodology employed to develop them [9–14]. Specifically, they should be developed through consensus methods involving stakeholder groups specific to the condition or intervention in question. There should be a strong emphasis on patient involvement in their development to ensure that the outcomes defined are personally important as well as clinically relevant [9].

Core outcome sets are likely to reduce the incidence of reporting bias, which arise because significant findings are more likely to be reported than those which are not significant [15]. Selective reporting of outcomes prevents informed decisions about treatment options and may have an adverse impact on the allocation of resources and research planning [16, 17].

The existence of a core outcome set does not preclude the investigation of other outcomes but rather it provides a minimum set that should be used in all research studies in the specific topic area. Thereby, it should improve consistency among trial reports and provide the valuable information needed to support meaningful systematic reviews and meta-analyses of similar studies.

Most core outcome sets have been developed to evaluate specific diseases or treatments, but we will develop one that is somewhat different. This core outcome set will be designed to evaluate interventions aimed at improving the consent process for people undergoing invasive procedures, rather than evaluating the effects of, for instance, those invasive procedures.

The scope of this study is restricted to interventions intended to improve consent in adults (over 18 years old) who have adequate mental capacity to make their own decisions regarding consent for surgery and invasive procedures, including endoscopy and dental surgery. The study has three main aims:

1. To identify a list of outcomes previously reported in trials of interventions aimed at improving consent for surgery and invasive procedures or suggested as relevant in articles discussing consent for treatment in general.
2. To identify additional outcome measures relevant to how interventions to improve consent are evaluated by exploring which outcomes matter to people involved in the consent process.
3. To define a core outcome set to evaluate interventions to improve consent for surgery and other invasive procedures through a Delphi survey and consensus meetings.

## Methods/design

**Fig. 1:**
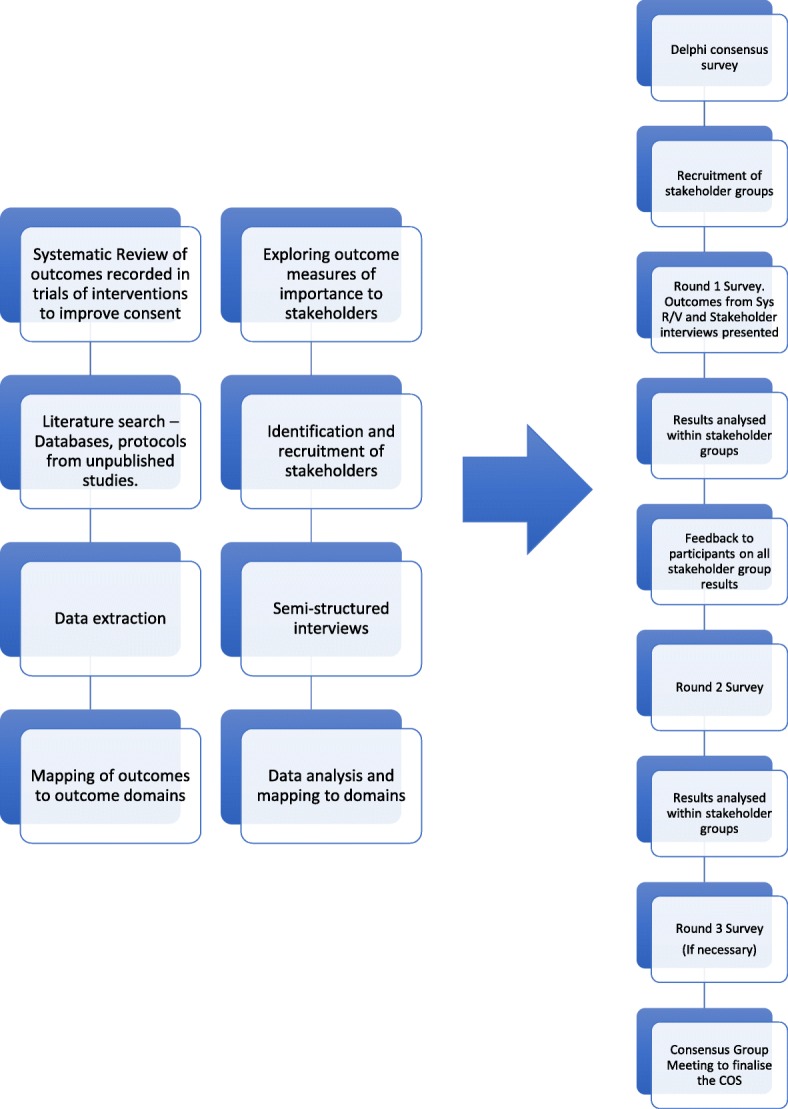
Flowchart for the Development of a Core Outcome Set to evaluate the effectiveness of interventions to improve informed consent for surgery

The development of this core outcome set will be conducted in accordance with guidelines and recommendations in the COMET handbook and the COS-STAD recommendations with reporting conforming with the Standard Protocol Items: Recommendations for Interventional Trials (SPIRIT) checklist. (See Additional File 1: SPIRIT Checklist) [9, 18]. The study will follow the steps outlined in Fig. 1.

### Systematic review

#### Criteria for included studies

Included studies will have attempted to investigate the effects of an intervention to improve or augment the consent process for surgery or other invasive procedures in adult patients with capacity to consent for themselves. Studies that have sought to identify the most effective way of evaluating the quality of consent will also be included.

Eligible studies will be meta-analyses, systematic reviews and randomised trials. Protocols for as yet unpublished studies will be included as well as full reports of these research studies. A sample of non-randomised controlled trials and prospective cohort studies will be reviewed for additional outcome measures, but a full systematic review of non-randomised quantitative research will not be conducted.

Qualitative studies using focus groups, surveys or interviews to determine which aspects are important in determining the quality of informed consent will be included in a separate meta-synthesis.

#### Exclusion criteria

We will exclude studies that focus on consent for interventions other than surgery or other invasive procedures. For example, we will not include studies investigating patients’ decisions to participate in research studies, as issues such as clinical equipoise and the therapeutic misconception make consent for participation in research and consent to treatment substantially different. Studies evaluating consent in patients without capacity or in children (under 18 years) will be excluded. Consent and assent are generally used to differentiate the legal competency of children over and under 16 years old, respectively, when they agree to clinical care. Parental permission is generally accompanied by assent, which indicates a child’s acquiescence, while consent infers a voluntary positive agreement. The consent process, information needs and decision-making processes for children differ significantly to those for adults consenting for themselves and thus, studies involving children will be excluded. Studies published in a language other than English will also be excluded.

#### Search strategy to identify studies

The systematic review will be undertaken and reported in accordance with the principles in the Preferred Reporting Items for Systematic Reviews and Meta-Analyses (PRISMA) statement [19].

A search strategy has been designed by a senior medical librarian and further refined through consultation with the principal investigator (LJC). The search for experimental studies will seek trials of interventions designed to standardise or improve the consent process for patients undergoing surgery or other invasive procedures. A separate search strategy will be developed for finding relevant qualitative studies.

Searches will made of MEDLINE (from 1946 to August 2017), EMBASE (1974 to August 2017), CINAHL (from 1937 to August 2017), CENTRAL (as of August 2017), PsycINFO (from 1806 to August 2017) and Web of Science (as of September 2017) using medical subject headings. Eligible ongoing and unpublished trials will be sought in ClinicalTrials.org and the WHO clinical trials registry platform (ICTRP) using the search term ‘informed consent’ [20, 21]. References in included studies will be checked to identify further relevant studies [22].

#### Eligibility of studies for inclusion

Two reviewers will screen the retrieved titles and abstracts to eliminate irrelevant studies. Full-text articles will be obtained for all studies considered to be potentially relevant from this initial screening. After reviewing the full text, any discrepancies in decisions about eligible studies will be resolved through discussion with a third senior reviewer.

#### Data extraction

The two independent reviewers will use a standardised recording template to extract data from the eligible studies. This will include study title, study author, study aim, type of study, study period, publication date, study location and number of participants. All primary and secondary outcomes, how the outcomes were measured (i.e. using a validated tool or otherwise), the rationale for the outcome measures chosen, the time point or period, and how outcomes were reported will all be recorded.

In the review of qualitative studies, each relevant full-text paper will be analysed independently by two authors. The following key details for each study will be recorded: year of publication, country in which the study was conducted, the phenomenon of interest, context, qualitative method used, number of participants and cultural setting. Furthermore, all funding sources and any potential conflicts of interest will be noted. Findings were defined as the main themes or findings detailed in the results section of each paper. Findings will be extracted verbatim from the text of the study with an accompanying illustrative quote where available. Each finding will be graded according to how confident the reader was in that finding. Unequivocal findings are those that are a matter of fact, beyond reasonable doubt, directly reported or observed, or not open to challenge. Credible findings are plausible interpretations of the primary data within the selected theoretical framework. However, since they are interpretations of the primary data, they are open to challenge. Finally, unsupported findings are those with no evidence from the primary data.

The methodological quality of the studies included will be assessed using the Cochrane risk of bias tool for randomised trials [23]. Qualitative studies will be evaluated using the Joanna Briggs Institute SUMARI software, using their criteria for appraising the quality of qualitative research [24]. Methodological quality is being assessed to facilitate the stratification of outcomes reported in studies at high and low risk of bias.

#### Data analysis

The data will be summarised in tabular form and coded according to what is measured. Agreement regarding outcome coding will be achieved between two researchers with a third senior researcher acting as an arbiter when necessary. We will repeat the coding of outcomes into broader outcome domains and constantly compare outcome information with these emerging domains until all outcomes have been assigned to an outcome category.

The findings of qualitative studies will be read repeatedly before being grouped into categories of similar findings. Category descriptions were defined through consensus among the authors. These categories were reviewed and refined using a constant comparison method. Following the classification of categories, synthesised findings combining two or more categories were agreed and developed through consensus among the authors.

The final structure and organisation of the outcome mapping process will be achieved through discussion within the research team, which includes experts in informed consent, taxonomy development and patient representation.

If an outcome was measured using a specific tool, details of that tool will be recorded verbatim to allow comparison with other tools designed to measure similar outcomes.

This process aims to catalogue comprehensively which outcomes have been measured in trials of interventions for informed consent for surgery in the form of an outcomes taxonomy. We also aim to incorporate the findings from a review of the qualitative literature into the taxonomy to facilitate the stakeholder consultation component of the research.

### Identifying items of importance to stakeholders

#### Stakeholders

Stakeholders with an interest in the consent process and patients will be invited to participate in semi-structured interviews to determine which outcomes each stakeholder group believe to be most important in determining the quality of consent.

People with expertise in informed consent and patients will be invited to participate in this phase of the research. It is anticipated that approximately 30 individuals from a wide range of backgrounds will be included. Proposed stakeholder groups include:

1. Patients
2. Clinicians with an interest in informed consent.
3. Solicitors practising medical law with particular reference to consent
4. Consent researchers
5. Bioethicists

A purposive sample of each stakeholder group will be identified and sampling will be performed within each stakeholder group to ensure there is a balance of participants across the groups. There will be approximately four to six individual interviews per stakeholder group. Participants from each group will be identified from the following sources:

1. Patients involved in qualitative research previously conducted by our group investigating the question ‘What is important to patients in the consent process?’ who have indicated that they would be happy to be involved in future research. These patients have undergone either emergency or elective surgery for a wide range of conditions, including minor day surgery and major surgery for benign and malignant conditions. Purposive sampling will ensure there is an adequate representation of patients from differing demographic groups and clinical backgrounds.
2. Non-patient groups will be identified through existing professional networks. If additional individuals are required, they will be identified through the following sources:
  - Clinicians with an interest in consent will be identified through the Association of Surgeons of Great Britain and Ireland.
  - Solicitors will be identified through the Law Society of Northern Ireland.
  - Consent researchers and bioethicists will be identified through the Society for Medical Decision Making and the Association of Bioethics, respectively, and through reviewing the contact details of published researchers.

A sample size of approximately 30 participants is in line with the interview component of other Delphi studies and will likely be sufficient to reach saturation. Stopping criteria will be applied within each stakeholder group. That is, three consecutive interviews with no new ideas contributed will indicate data saturation. These techniques have been demonstrated to be appropriate for use in qualitative research and they ensure that the data are representative of the population being studied whilst not wasting resources by continuing to conduct interviews when no new ideas are being presented [25].

Purposive sampling will ensure there is a diverse range of participants so that a variety of perspectives are captured on what is important in determining the quality of informed consent.

Potential participants will be contacted by email to provide study information and to invite them to participate in the interview. Interested individuals will be asked to contact the investigators to arrange a convenient time to conduct the interview by telephone or Skype.

Informed consent will be obtained using a standardised script before commencing each interview. Telephone and Skype interviews have been chosen over face-to-face interviews to minimise inconvenience for participants and to facilitate the inclusion of individuals over a wide geographic area. These interviews will be audio-recorded and contemporaneous notes will be kept by the interviewer.

#### Conduct of semi-structured interviews

Participants will not be provided with a summary of the findings from the systematic review prior to the interview as we do not wish to focus the participants’ attention on previously reported outcomes. Definitions with explanatory notes for each of the outcomes identified will be developed by the research team and supplied to participants to improve their understanding.

Specifically, participants will be asked to comment on the relative importance of specific outcomes in determining the quality of informed consent. They will be asked to suggest additional outcomes that may be of relevance and how these may be measured. This will allow identification of as yet unstudied outcomes. Participants will be asked which outcomes they believe to be core.

#### Qualitative data analysis

The analysis of the interviews will begin during the data collection, to help refine and develop future interviews. Two reviewers will independently assess the transcripts to identify key points that stakeholders believe to be important in the consent process through a constant comparison approach [26].

When the dataset is complete, qualitative data will be analysed within stakeholder groups. The aim of the analysis will be to define which outcomes are deemed to be most important and explore the reasons why people believe they are important.

The data will be analysed using a framework approach, which is an established qualitative analysis method that employs constant comparison techniques and grounded theory [26–28]. The framework method involves the analyst familiarising themselves with the data, developing a thematic framework, indexing the data, mapping the data to the framework and finally interpreting the results [27, 29]. A second investigator will review the themes identified.

Ethical approval for the qualitative interviews with stakeholders has been obtained from the regional ethics committee (Office for Research Ethics Committees Northern Ireland reference: RECA 17/NI/0234).

### Developing a core outcome set

A Delphi consensus survey will be used to establish which outcomes are most important to stakeholders involved in the informed consent process. The Delphi consensus model allows participants to respond without being influenced by others and will facilitate analysis within stakeholder groups. All participants will have equal weighting. The process also allows participants to complete the survey at a time that is convenient for them and is not bound by geographical or time zone constraints.

#### Outcomes included for consensus exercise

The list of outcomes derived from the systematic review and the semi-structured interviews will be listed individually within the predetermined outcome domains agreed by the research group. The survey will be reviewed by the research group for comprehension and pilot tested to ensure readability and the usability of the online tool.

We will conduct a series of cognitive interviews using a think aloud technique among a group of patient volunteers. The purpose of the cognitive interviews will be to ensure patients know how to complete the survey, comprehend the meaning of the items and understand how to use the 9-point response scale. Interviews will be conducted in accordance with best practice in this field [30, 31]. Participants will be asked open-ended questions to:

- Gauge their understanding of the meaning of each item in the survey
- Understand the clarity of each item from the patient’s perspective
- Learn how patients interpret the response options
- Identify patient difficulties with the format of the survey
- Determine the length of time it takes to complete the survey.

The interviews will be video-recorded and notes on patient body language recorded as they review the Delphi survey. After the first five interviews, the survey will be amended in accordance with patient feedback, with a further five interviews being subsequently conducted to determine if any further changes are necessary.

#### Identification and recruitment of participants

Stakeholders will be invited to participate in the study via email. A gatekeeper on behalf of the research team will contact potential participants. Participants will be identified and contacted through the following sources:

1. Our existing database of patients who have expressed interest in participating in future work.
2. Patients who respond to our invitation on the National Institute for Health Research’s People in Research website (https://www.peopleinresearch.org/)
3. Non-patient groups will be approached through the research group’s professional networks in the first instance. If additional participants are required, they will be identified as follows:
  - Clinicians with an interest in consent will be identified through the Association of Surgeons of Great Britain and Ireland.
  - Solicitors will be identified through the Law Society of Northern Ireland.
  - Consent researchers and bioethicists will be identified through the Society for Medical Decision Making and the Association of Bioethics, respectively, and by reviewing the contact details of published researchers. This group will also contain participants from a legal background who have published on informed consent from jurisdictions other than the United Kingdom to include an international legal perspective.

Invitations to participate in the Delphi survey will be sent by email. Those who are willing to participate can click on a link in the email that will lead to the online Delphi survey. Participants will be asked to register their details and identify which stakeholder group they belong to. This will generate a unique ID for each participant. The system facilitates the linking of participant responses with their unique ID and will send reminder emails at the end of week 2 of each 3-week round if necessary.

Consent for completion of the Delphi survey will be implied by completion of the questionnaire. Participants will, however, be asked if they would be willing to participate in a future web-based consensus agreement meeting. Ethical approval for the Delphi survey will be obtained.

#### The Delphi survey

Participants will be asked to consider the following question:

*Think about the process of consent for surgery. How important do you think each of the items below would be in telling you if the process had been performed well?*

We will use COMET Delphi manager, a web-based tool designed specifically for Delphi surveys by the COMET initiative, to ask participants to score the importance of the listed outcome measures on a 9-point Likert scale. The scale will be labelled to indicate that a score of 1 to 3 is of ‘limited importance’, a score of 4 to 6 is ‘important but not critical’ and a score of 7 to 9 is of ‘critical importance’ [32]. A definition of each outcome will be provided to aid participant comprehension.

### Round 1

In addition to being asked to rate the importance of all the outcomes, participants will be given the opportunity in round 1 to suggest additional outcomes that had not been identified previously.

Descriptive statistics will be used to summarise the results. The frequency distribution, median and interquartile ranges will be calculated from the responses of each stakeholder group for each outcome. All outcomes that are included in the initial list for round 1 will be carried forward to round 2. Any new additional outcomes will be reviewed and coded by two investigators to ensure they are independent of the outcomes already listed.

The number of participants starting the survey, the number completing the survey and the number of non-respondents will be recorded.

### Round 2

Round 2 assumes that adequate numbers responded to round 1. In this study, we wish to have at least 10 participants per stakeholder group before proceeding, which has been deemed acceptable in previous work [14]. If the minimum number of responders is not achieved, this will be discussed by the research group.

The survey for round 2 will present participants with feedback from all stakeholder groups combined, for each outcome from round 1. These data will be presented graphically demonstrating the full spread of scores and reminding participants of their own response in the previous round before asking them again to rate the importance of each outcome on the 9-point scale in the context of the feedback they have received. Participants will also be given the opportunity to state whether and why each outcome should be included in the final core outcome set and to explain why they changed their score from round 1, if they did so.

There is evidence to support the provision of feedback from all stakeholder groups in terms of improving consensus, reducing the variability of responses and improving agreement on those items to keep at the conclusion of the process [33].

The number of participants completing round 2 will be recorded and analysed for evidence of response bias between the round 1 and round 2 feedback groups. Descriptive statistics will be used to summarise the responses to each outcome.

A predetermined standard of 70% of respondents rating an outcome 7 or higher and less than 15% rating it 3 or lower will rule an outcome in for inclusion in the core outcome set. Likewise, outcomes with less than 15% of respondents rating it at least 7 and greater than 70% rating it 3 or lower will be excluded from the core outcome set. Outcomes not meeting these criteria will be classified as equivocal and will require further work (as detailed below). These standards are in keeping with a growing body of core outcome set literature [10, 13, 14, 34]. Although the criteria are somewhat subjective, no defined rules exist for inclusion or exclusion, and bias will be minimised by determining the inclusion and exclusion thresholds at the protocol stage.

### Round 3

It is hoped that adequate consensus can be achieved during the first two rounds of the Delphi survey so that the list of outcomes has been distilled down to a manageable number to take forward to the consensus meetings. If this is not the case, a third round of the Delphi survey will be undertaken. Participants will be presented with the distribution of scores for each outcome for each of the stakeholder groups and reminded of their personal score from round 2. Participants will then be asked to rescore all outcomes and consider whether, and why, they should be included in a core outcome set. The methodology for this round will follow the steps outlined in round 2.

### Patient participant focus groups

Following completion of the Delphi survey, patients who have indicated a willingness to be involved in additional aspects of the research will be invited to participate in a focus group. They will be shown the findings of the Delphi survey and their views on those findings elicited. Specifically, the research team will ask them to express their views on the importance of those outcomes that were deemed equivocal from the Delphi process and to confirm whether they believe that the outcomes that met the criteria for definite inclusion and exclusion from the core outcome set were classified appropriately.

Patients will not be included in the subsequent consensus webinars, as previous consensus-building exercises have found that laypeople are reluctant to contribute fully in a discussion with perceived experts in such settings. However, the results from the patient focus group will be presented to the participants of the consensus webinars.

### Web-based consensus meetings

The main objective of the web-based consensus meeting will be to determine whether to include or exclude outcomes that were equivocal in the survey portion of the study.

Participants will have been identified in earlier rounds of the research as being willing to participate in a consensus meeting. Informed consent will be obtained. The research team will facilitate the webinars. The meeting dialogue will be audio-recorded using Adobe Connect Pro software (Adobe systems Incorporated, San Jose, CA).

The primary focus of the webinars will be to decide whether equivocal outcomes should be included in the final core outcome set. However, there will also be an opportunity for the participants to review the outcomes already excluded and included, to ensure any minority opinions can be documented.

A final core set of what outcomes to measure to determine the effects of interventions to improve informed consent will be formulated following the two webinars. Determining how and when to record outcomes is beyond the scope of this research. The lack of a tool to measure any individual outcome will not preclude its inclusion in the core outcome set.

Two separate webinars will be conducted involving different participants. This will:

1. Give me flexibility in the time and date of the webinars to encourage participation.
2. Allow meetings at different times to facilitate participation from stakeholders in different time zones (e.g. North America and Australasia).
3. Allow comparison between the core outcome sets generated by these independent consensus webinars.

The research team facilitating the second webinar will not reference the outcome of the first webinar during it.

The final core outcome set and its development will be reported in line with COS-STAR guidance [12].

### Statistical considerations

All data will be collected prospectively and entered into a secure electronic database by members of the research team.

No standardised method of calculating the sample size for Delphi studies exists. However, there is evidence to suggest that panels of approximately 20 people can produce stable results [35]. Therefore, to allow for 50% attrition between rounds, we will aim to recruit a minimum of 40 participants per stakeholder group in round 1.

## Discussion

Currently, no core outcome set exists for the evaluation of tools designed to improve the quality of informed consent for surgery or other invasive procedures. The ICONS study aims to fill this gap. We hope that this core outcome set will reduce the level of selection and reporting bias in consent research and help clinicians to compare tools to improve consent so that patients are better informed. By ensuring key stakeholder involvement in all stages of the development process, we hope that the core outcome set will be fit for purpose and will be well accepted in future research internationally.

### Trial status

This protocol does not describe a trial. The protocol outlines the development of a core outcome set to be used in future trials of interventions to improve informed consent for surgery and other invasive procedures. This study is currently in the set-up phase.

## Additional file

Additional file 1: SPIRIT 2013 Checklist: Recommended items to address in a clinical trial protocol and related documents. (DOC 121 kb)

## Abbreviation

COMET: Core Outcome Measures in Effectiveness Trials
